# Stress Hyperglycemia Ratio as a Predictor of All‐Cause and Cardiovascular Mortality in the General Population: Insights From NHANES 2001–2018

**DOI:** 10.1155/ije/9929815

**Published:** 2026-04-22

**Authors:** Lei Zhang, Chang Shu, WenTao Hu, WanCheng Guo, PengCheng Guo, Rui Li, Dexiang Xia, Xin Li

**Affiliations:** ^1^ Department of Vascular Surgery, The Second Xiangya Hospital, Central South University, Changsha, China, csu.edu.cn; ^2^ Angiopathy Institute of Central South University, Changsha, China; ^3^ Clinical Research Center for Vascular Disease in Hunan Province, Changsha, Hunan, China; ^4^ Department of Cardiovascular Surgery, Chinese Academy of Medical Sciences and Peking Union Medical College Fuwai Hospital, Xicheng District, Beijing, China; ^5^ Department of Vascular Surgery, Guilin Hospital of the Second Xiangya Hospital, Central South University, Guilin, China, csu.edu.cn; ^6^ Department of Urology, The Second Xiangya Hospital, Central South University, Changsha, China, csu.edu.cn

**Keywords:** ACM, CVM, NHANES, SHR

## Abstract

**Background:**

Stress hyperglycemia ratio (SHR) is closely related to adverse clinical outcomes. However, the link between general population all‐cause mortality (ACM) and cardiovascular mortality (CVM) remains undetermined. Thus, this research was designed to investigate the associations between SHR and ACM and CVM.

**Methods:**

The research data are sourced from NHANES. The correlations between SHR and death were examined via multivariate Cox proportional hazards models. These models were adjusted for clinical, socioeconomic, and demographic characteristics. Potential nonlinear relationships were estimated via restricted cubic spline (RCS) analysis. Predictive accuracy was measured by time‐dependent ROC curves, and mortality risk across SHR quartiles was examined by Kaplan–Meier survival curves. Age, sex, race, BMI, level of education, smoke, and poverty‐income ratio were used as subgroups to investigate potential moderators of the effect.

**Results:**

About 1847 people died from any cause and 589 from cardiovascular disease throughout the median follow‐up period of 97 months. SHR was negatively associated with ACM (HR: 0.77, 95% CI: 0.68–0.88) and CVM (HR: 0.73, 95% CI: 0.60–0.90) when SHR was below the threshold values (SHR < 0.81 in ACM and SHR< 0.84 in CVM), whereas it was positively associated with ACM (HR: 1.10, 95%CI: 1.05–1.15) and CVM (HR: 1.08, 95%CI: 1.00–1.16) when it was above the threshold values. The ROC curve revealed that the SHR may serve as a predictor of ACM and CVM.

**Conclusions:**

SHR exhibits a nonlinear, threshold‐dependent association with ACM and CVM, and it may serve as a predictor of ACM and CVM.

## 1. Background

Glycemic dysregulation during stress has become a crucial factor influencing clinical outcomes in several chronic and acute diseases. Historically, fasting plasma glucose (FPG) and glycated hemoglobin (HbA1c) have functioned as essential biomarkers for evaluating short‐term and long‐term glucose regulation, respectively. Nonetheless, neither adequately reflects the magnitude of acute glucose rise in comparison to baseline glycemia during physiological or pathological stress. The stress hyperglycemia ratio (SHR), a novel metric that integrates admission FPG with HbA1c, provides a more thorough evaluation of acute glycemic variations by considering both present and past glycemic conditions [1]. Consequently, SHR indicates an individual’s glycemic reaction to stress and is widely acknowledged as a potential indicator of metabolic instability. Multiple investigations illustrated a noticeable link between elevated SHR and worse clinical outcomes in populations subjected to acute stress. SHR has been related to elevated cardiovascular mortality (CVM) in individuals with acute coronary syndrome [2, 3]. Multicenter research revealed a J‐shaped connection between SHR and ACM in individuals with acute myocardial infarction [4]. Individuals with heart failure and concurrent sepsis have shown the SHR to be an independent prognostic factor; individuals with ischemic stroke have shown a strong correlation between the SHR and the pathogenesis of cerebral edema and low functional outcomes [5, 6]. Ischemic stroke individuals with SHR had an elevated risk of death from both immediate and delayed complications, and this was true even after controlling for diabetes [7]. There is a strong link between the SHR and in‐hospital death in individuals with septic shock [8].

The correlation between SHR and overall death risk has not been adequately studied in general population. This study aims to use data from a large, representative U.S. population to examine the correlation between SHR and CVM and ACM. We hypothesize that SHR is associated with mortality outcomes in a nonlinear fashion and could be a good indicator of the likelihood of dying in the future. By extending the predictive usefulness of SHR beyond specific illness populations and into the general population, this investigation hopes to increase the therapeutic significance of SHR and improve risk stratification strategies at the population level.

## 2. Methods

### 2.1. Study Population and Design

Through the use of information gathered from the National Health and Nutrition Examination Survey (NHANES), a program run every 2 years by the National Center for Health Statistics (NCHS), an arm of the CDC in the US, this study conducted a population‐based observational analysis. Health and nutrition data that are representative of the noninstitutionalized civilian population of the US are gathered by NHANES using a multistage, stratified, probability‐cluster sampling process. An initial sample of 91,351 people was used to gather data from the 2001–2018 survey cycles from the official NHANES repository.

**FIGURE 1:**
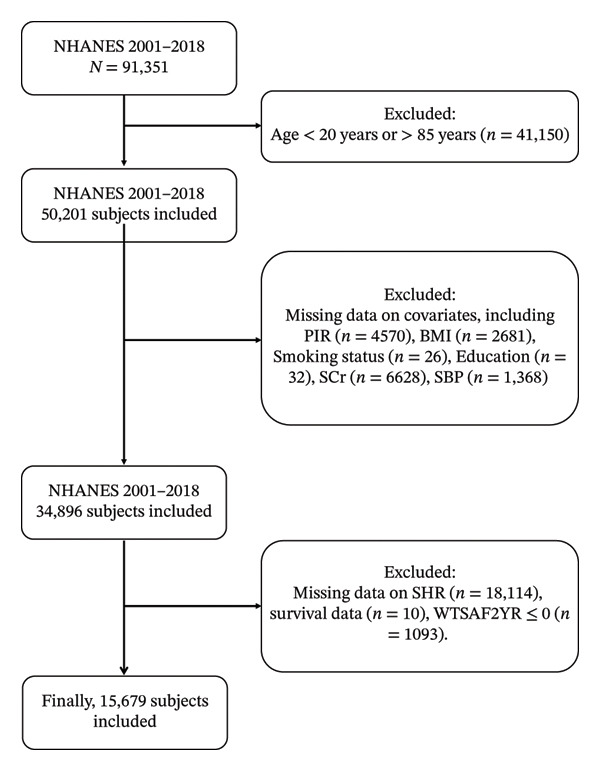
Sample selection flow from NHANES 2001–2018.

Participants were disqualified if they fulfilled any of the subsequent criteria: (1) age less than 18 or greater than 85 years; (2) incomplete data for essential covariates (age, sex, ethnicity, body mass index (BMI), education, smoking status, family poverty income ratio [PIR]); (3) absence of FPG or HbA1c measurements necessary for SHR calculation; (4) unavailability of connected mortality data; and (5) fasting subsample weight (WTSAF2YR) less than or equal to 0. Following exclusions, 18,384 individuals were included (Figure 1).

### 2.2. SHR Calculation

SHR = (FPG [mmol/L])/(1.59 ∗ HbA1c [%] −2.59). The participants were classified into four groups, with Q1 being used as the reference group for the following analyses, according to the SHR distribution.

### 2.3. Mortality Outcomes

The CVM included deaths from cardiovascular or cerebrovascular causes, while ACM encompassed deaths from any cause, classified using ICD‐10 codes.

#### 2.3.1. Assessment of Covariates

Sociodemographic and clinical factors in this study were extracted from NHANES interviews and laboratory records. Covariates encompassed age, sex, race/ethnicity, BMI, educational attainment, smoking status, and PIR. The history of chronic diseases was derived from self‐reported physician diagnoses. Diabetes was classified based on established diagnostic criteria: self‐reported diabetes, administration of insulin or oral hypoglycemics, FPG (FPG) ≥ 7.0 mmol/L, 2‐h oral glucose tolerance test (OGTT) ≥ 11.1 mmol/L, or HbA1c ≥ 6.5%. Prediabetes is characterized by self‐reported prediabetes or FPG levels of 5.6–6.9 mmol/L, a 2‐h OGTT result of 7.8–11.0 mmol/L, or hemoglobin A1c (HbA1c) levels (5.7%–6.4%).

Lab markers such as total protein, albumin, alanine aminotransferase (ALT), as well as aspartate aminotransferase (AST) were collected to be used for sensitivity analysis adjustments when needed.

### 2.4. Statistical Analysis

Considering the complexity of the NHANES sampling design, this study incorporated sample weights, clustering, and stratification into the analysis. This study process considered sample weights and defined the new weights as the weight of fasting subsample (WTSAF2YR) divided by 9.

Weighted descriptive analyses: The SHR quartiles (Q1–Q4) were used to report descriptive data. All descriptive statistics presented in Tables 1 and 2 were weighted to produce nationally representative estimates. The groups were compared via one‐way analysis of variance (ANOVA) with continuous variables given as mean ± SD. Pearson’s chi‐squared tests were utilized to observe categorical variables, which were reported as counts (proportions).

**TABLE 1:** Baseline characteristics stratified by SHR quartiles in general population.

SHR_quart							
Characteristic	*N*	Overall, *N* = 15,679 (100%)	Q1, *N* = 3920 (21%)	Q2, *N* = 3955 (26%)	Q3, *N* = 3893 (26%)	Q4, *N* = 3911 (27%)	*p* value
Age (years)	15,679	47.0 (16.8)	49.1 (16.9)	46.9 (16.7)	45.9 (16.7)	46.6 (16.7)	**< 0.001**
Sex	15,679						**< 0.001**
Female		8045 (51%)	2347 (62%)	2223 (58%)	1902 (49%)	1573 (39%)	
Male		7634 (49%)	1573 (38%)	1732 (42%)	1991 (51%)	2338 (61%)	
Race	15,679						**< 0.001**
Mexican American		2529 (8.2%)	539 (7.2%)	618 (7.8%)	700 (9.2%)	672 (8.3%)	
Other Hispanic		1348 (4.9%)	314 (4.7%)	345 (5.1%)	342 (5.2%)	347 (4.4%)	
Non‐Hispanic White		7128 (69%)	1439 (60%)	1814 (69%)	1889 (71%)	1986 (74%)	
Non‐Hispanic Black		3035 (11%)	1223 (20%)	727 (10%)	540 (7.2%)	545 (6.9%)	
Other Race		1639 (7.3%)	405 (7.6%)	451 (7.6%)	422 (7.4%)	361 (6.6%)	
BMI, kg/m^2^	15,679						**< 0.001**
Normal (18.5–< 25)		4337 (29%)	1156 (32%)	1199 (33%)	1027 (28%)	955 (24%)	
Obese (30 or greater)		5867 (36%)	1465 (36%)	1365 (33%)	1437 (36%)	1600 (41%)	
Overweight (25 to < 30)		5239 (33%)	1228 (30%)	1320 (32%)	1385 (35%)	1306 (34%)	
Underweight (< 18.5)		236 (1.5%)	71 (1.8%)	71 (2.0%)	44 (1.2%)	50 (1.1%)	
Education	15,679						**0.001**
Less than 9th Grade		1596 (5.3%)	429 (6.3%)	370 (4.7%)	390 (5.4%)	407 (5.2%)	
9–11th Grade		2195 (11%)	582 (13%)	510 (9.8%)	505 (9.4%)	598 (11%)	
High School Grad/GED		3610 (23%)	940 (24%)	892 (23%)	893 (23%)	885 (24%)	
Some College or AA degree		4604 (31%)	1129 (30%)	1199 (32%)	1154 (32%)	1122 (30%)	
College Graduate or above		3674 (29%)	840 (27%)	984 (30%)	951 (30%)	899 (31%)	
PIR	15,679						**0.002**
[1, 3)		6638 (37%)	1700 (38%)	1654 (36%)	1641 (36%)	1643 (37%)	
[3, Inf)		5864 (50%)	1369 (46%)	1549 (51%)	1464 (50%)	1482 (51%)	
[0, 1)		3177 (14%)	851 (16%)	752 (13%)	788 (14%)	786 (12%)	
Smoking status	15,679						**< 0.001**
Never smoker		8578 (54%)	2182 (55%)	2205 (54%)	2169 (55%)	2022 (53%)	
Former smoker		3968 (25%)	904 (23%)	980 (25%)	948 (25%)	1136 (28%)	
Current smoker		3133 (20%)	834 (23%)	770 (21%)	776 (20%)	753 (19%)	
SBP	15,679	121 (17)	122 (18)	120 (17)	120 (16)	123 (17)	**< 0.001**
eGFR	15,679	97 (21)	94 (22)	97 (20)	98 (20)	98 (20)	**< 0.001**
HTN_med	15,679	4261 (23%)	1144 (25%)	952 (21%)	984 (22%)	1181 (26%)	**< 0.001**
lipid_med	15,679	3271 (19%)	876 (19%)	768 (19%)	751 (18%)	876 (20%)	0.3
CVD.group	15,679						**0.029**
CVD		1721 (8.8%)	470 (9.8%)	399 (7.7%)	391 (8.3%)	461 (9.3%)	
non‐CVD		13,958 (91%)	3450 (90%)	3556 (92%)	3502 (92%)	3450 (91%)	
diabetes.group	15,679						**< 0.001**
diabetes		3094 (15%)	786 (16%)	472 (8.6%)	562 (10%)	1274 (24%)	
normal		6069 (44%)	1538 (47%)	2142 (60%)	1579 (47%)	810 (24%)	
prediabetes		6516 (41%)	1596 (37%)	1341 (31%)	1752 (43%)	1827 (52%)	
Cancer.group	15,679						0.4
Cancer		1460 (9.3%)	379 (10%)	375 (9.3%)	330 (8.7%)	376 (9.3%)	
Noncancer		14,219 (91%)	3541 (90%)	3580 (91%)	3563 (91%)	3535 (91%)	
ACM	15,679	1847 (8.4%)	502 (10%)	397 (6.8%)	422 (7.8%)	526 (9.3%)	**< 0.001**
CVM	15,679	591 (2.5%)	175 (3.3%)	122 (2.0%)	135 (2.3%)	159 (2.7%)	**0.016**
SHR	15,679	0.93 (0.12)	0.78 (0.06)	0.88 (0.02)	0.95 (0.02)	1.08 (0.11)	**< 0.001**

*Note:* PIR, family income‐poverty ratio. The bold values indicate statistically significant differences (*p* < 0.05).

Abbreviations: ACM, all‐cause mortality; BMI, body mass index; CVM, cardiovascular mortality; eGFR, estimated glomerular filtration rate; SHR, stress hyperglycemia ratio.

**TABLE 2:** Baseline laboratory characteristics stratified by SHR quartiles in general population.

SHR_quart							
Characteristic	*N* ^1^	Overall, *N* = 15,679 (100%)^2^	Q1, *N* = 3920 (21%)^2^	Q2, *N* = 3955 (26%)^2^	Q3, *N* = 3893 (26%)^2^	Q4, *N* = 3911 (27%)^2^	*p* value^3^
TP (g/L)	15,668	70.97 (4.50)	70.92 (4.83)	70.87 (4.41)	71.05 (4.45)	71.03 (4.37)	0.2
Albumin (g/L)	15,679	42.29 (3.38)	41.59 (3.50)	42.22 (3.26)	42.50 (3.27)	42.71 (3.42)	**< 0.001**
ALT (U/L)	15,661	25.28 (19.37)	23.88 (24.16)	24.03 (18.77)	24.92 (16.10)	27.93 (18.34)	**< 0.001**
AST(U/L)	15,651	25.07 (18.34)	25.29 (22.99)	24.24 (21.22)	24.70 (12.95)	26.07 (15.48)	**< 0.001**
ALP (U/L)	15,676	67.97 (24.04)	68.85 (26.47)	66.76 (21.86)	67.12 (22.30)	69.27 (25.53)	**0.004**
TBIL (umol/L)	15,670	12.12 (5.31)	11.54 (4.71)	11.79 (5.11)	12.15 (5.33)	12.88 (5.82)	**< 0.001**
GGT (U/L)	15,677	27.74 (37.15)	26.49 (31.08)	24.44 (26.26)	27.00 (32.80)	32.61 (51.31)	**< 0.001**
TG (mmol/L)	15,669	1.46 (1.33)	1.34 (0.96)	1.36 (0.96)	1.43 (1.39)	1.67 (1.73)	**< 0.001**
Cholesterol (mmol/L)	15,676	5.04 (1.09)	5.09 (1.14)	5.09 (1.06)	5.03 (1.08)	4.96 (1.07)	**< 0.001**
Cr (μmol/L)	15,679	77.95 (31.03)	78.98 (41.85)	76.46 (26.23)	77.26 (30.08)	79.24 (25.56)	**< 0.001**
BUN (mmol/L)	15,677	4.75 (1.89)	4.77 (2.16)	4.66 (1.78)	4.69 (1.71)	4.89 (1.92)	**< 0.001**
UA (μmol/L)	15,675	325.18 (82.94)	316.74 (87.02)	317.17 (79.98)	326.89 (80.81)	337.83 (82.82)	**< 0.001**
Na+ (mmol/L)	15,679	139.31 (2.27)	139.32 (2.23)	139.31 (2.19)	139.28 (2.25)	139.34 (2.38)	> 0.9
K+ (mmol/L)	15,676	4.03 (0.33)	4.03 (0.34)	4.04 (0.33)	4.04 (0.32)	4.03 (0.35)	0.5
Ca2+ (mmol/L)	15,663	2.34 (0.09)	2.34 (0.09)	2.34 (0.08)	2.34 (0.09)	2.35 (0.09)	0.4

*Note:* ALP, alkaline phosphatase; ALT, alanine aminotransferase; AST, aspartate aminotransferase; Cr, creatinine; TBIL, total bilirubin; TG, triglycerides. The bold values indicate statistically significant differences (*p* < 0.05).

Abbreviations: BUN, blood urea nitrogen; GGT, gamma‐glutamyl transferase; TP, total protein; UA uric acid.

^1^ *N* not missing (unweighted).

^2^Mean (SD) for continuous variables; *n* (%) for categorical variables.

^3^Design‑based Kruskal–Wallis test.

Three multivariate Cox proportional hazards regression models were created to investigate the relationships between SHR and death outcomes. Model 1: Unadjusted, Model 2: adjusted for age, sex, and race, and Model 3: adjusted for age, sex, race, BMI, education, smoking, and family PIR, cardiovascular diseases, diabetes, cancer, blood pressure–lowering drugs, lipid‐lowering drugs, systolic blood pressure (SBP), and estimated glomerular filtration rate (eGFR). Four‐knot restricted cubic splines (at 5th, 35th, 65th, 95th percentiles, selected by AIC/BIC) were used to examine nonlinear SHR–mortality associations. Inflection points were defined as the SHR value where the predicted HR crossed 1. To assess the robustness of the identified inflection points, we performed sensitivity analyses by varying the number of knots (3–6) and knot placements, as well as using different covariate adjustment sets. The stability of the inflection points was further evaluated using bootstrap resampling with 1000 replicates. Upon detecting nonlinearity, inflection points were discovered, and a two‐piecewise Cox regression model was employed to estimate relationships both below and above the threshold. A Kaplan–Meier survival analysis was conducted for SHR segments delineated by inflection points. Time‐dependent ROC curves assessed SHR’s prediction accuracy for mortality outcomes over temporal intervals.

The sample was divided into subgroups based on their age (< 60 or ≥ 60 years), sex, race, BMI, level of education, tobacco consumption, and PIR classifications. This investigation’s statistical analyses were carried out via R (v 4.4.3). *p* < 0.05 was significant.

## 3. Results

### 3.1. Baseline Characteristics by SHR Quartiles

This study included 15,679 participants with a mean age of 46.7 years, of whom 8045 (51%) were female. The mean SHR was 0.93 ± 0.12. Compared with the participants in Q1, the participants in the study with a higher SHR (Q2–Q4) might be younger obese men, a greater proportion of non‐Hispanic white, and a greater proportion of smokers (Table 1). In the analysis of laboratory indicators, the research participants with a higher SHR (Q2–Q4) had higher laboratory indicators such as albumin, ALT, and AST (Table 2).

### 3.2. Relationship Between SHR and Death

After a median follow‐up period of 97 months for 15,679 study participants, 1847 (11.8%) patients experienced all‐cause mortality, of which 591 (3.8%) was attributed to cardiovascular‐related deaths (Table 3). The outcomes of the multivariate analysis are summarized in Table 3. For the reference group Q1, the fully adjusted model (Model 3) found a HR of 0.80 (95% CI: 0.65–0.95) for Q2, a hazard ratio (HR) of 0.96 (95% CI: 0.81–1.14) for Q3, and a HR of 1.11 (95% CI: 0.93–1.32) for Q4. The *p* for trend was 0.089. For CVM, the HRs were 0.74 (95% CI: 0.57–0.97) for Q2, 0.86 (95% CI: 0.62–1.21) for Q3, and 0.91 (95% CI: 0.68–1.20) for Q4, with a *p* for trend of 0.773.

**TABLE 3:** Cox regression modeling of the link between SHR index quartiles and death under different models.

**Quartiles of SHR index**					
	**Q1**	**Q2**	**Q3**	**Q4**	**p** **for trend**

All‐cause mortality					
Number of deaths	502/3920	397/3955	422/3893	526/3911	
Model 1 HR (95% CI)	1	0.67 (0.57, 0.79)	0.78 (0.65, 0.94)	1.01 (0.85, 1.20)	0.493
*p* value		< 0.001	0.008	0.9	
Model 2 HR (95% CI)	1	0.75 (0.63, 0.88)	0.91 (0.75, 1.10)	1.14 (0.97, 1.35)	0.031
*p* value		< 0.001	0.3	0.11	
Model 3 HR (95% CI)	1	0.80 (0.65, 0.95)	0.96 (0.81, 1.14)	1.11 (0.93, 1.32)	0.089
*p* value		0.008	0.7	0.2	
CV mortality					
Number of deaths	175/3920	122/3955	135/3893	159/3911	
Model 1 HR (95% CI)	1	0.60 (0.45, 0.81)	0.73 (0.51,1.05)	0.90 (0.68, 1.20)	0.822
*p* value		< 0.001	0.088	0.5	
Model 2 HR (95% CI)	1	0.69 (0.51,0.91)	0.86 (0.60, 1.24)	1.04 (0.78, 1.38)	0.500
*p* value		0.010	0.4	0.8	
Model 3 HR (95% CI)	1	0.74 (0.57, 0.97)	0.86 (0.62, 1.21)	0.91 (0.68, 1.20)	0.773
*p* value		0.030	0.4	0.5	

*Note:* Model 1: no covariates were adjusted for. Model 2: adjusted for age, sex, and race. Model 3: adjusted for age, sex, race, body mass index, education, smoking, and family PIR, cardiovascular diseases, diabetes, cancer, blood pressure–lowering drugs, lipid‐lowering drugs, systolic blood pressure, eGFR.

Abbreviations: CI, confidence interval; HR hazard ratio.

### 3.3. SHR–Mortality Nonlinearity

Possible nonlinear relationships between SHR and mortality were indicated by multivariate Cox proportional hazards regression models. To delve more into this connection, researchers utilized a 4‐knot regression model in an RCS that took into account age, sex, race, BMI, education, smoking, and PIR.

A strong nonlinear correlation was found between the SHR and both ACM (*p* < 0.0001) and CVM (*p* = 0.0004) after controlling for variables. Figure 2(a) shows the ACM inflection point at 0.81, and Figure 2(b) shows the CVM inflection point at 0.84, both determined by the RCS curves. After adjusting for age, sex, race, BMI, education, smoking, PIR, cardiovascular diseases, diabetes, cancer, blood pressure–lowering drugs, lipid‐lowering drugs, SBP, and eGFR, a two‐piecewise Cox regression model revealed that below the inflection point (SHR < 0.81 for ACM and < 0.84 for CVM), each 0.1‐unit increase in SHR was associated with a significantly reduced risk of ACM (HR = 0.77; 95% CI: 0.68–0.88) and CVM (HR = 0.73; 95% CI: 0.60–0.90) (Table 4). Above the threshold, the association reversed, with each 0.1‐unit increase in SHR being associated with a significantly higher risk (ACM: HR = 1.10, 95% CI: 1.05–1.15; CVM: HR = 1.08, 95% CI: 1.00–1.16) (Table 4). Among participants with SHR below the ACM inflection point (SHR < 0.81), there were 340 deaths (14.2%) among 2389 individuals; for those with SHR ≥ 0.81, 1507 deaths (11.3%) occurred among 13,290 individuals. For CVM, below the threshold (SHR < 0.84), there were 173 CV deaths among 3766 participants (4.6%), and above the threshold (SHR ≥ 0.84), 418 CV deaths among 11,913 participants (3.5%). These event counts confirm sufficient sample size on both sides of each threshold, supporting model stability.

FIGURE 2Adjusted non‐linear associations between SHR and death in the general population. (a) ACM; (b) CVM. Models adjusted for age, sex, ethnicity, BMI, education, smoking, and family PIR. Solid lines indicate estimated hazard ratios (HRs); red areas show 95% confidence intervals (CIs). Inflection points: SHR = 0.81 (a) and SHR = 0.84 (b). CVM 1.13 1.17.

**Figure:**
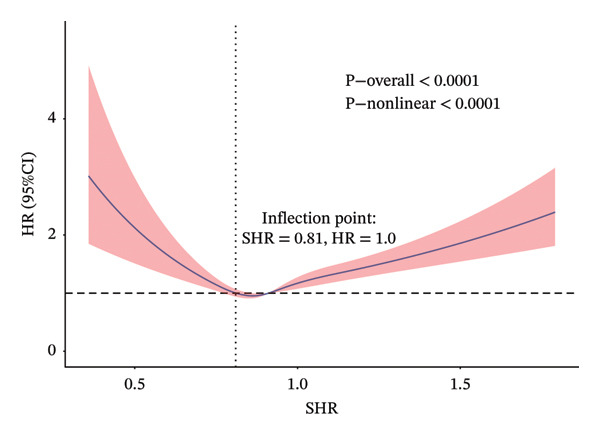
(a)

**Figure:**
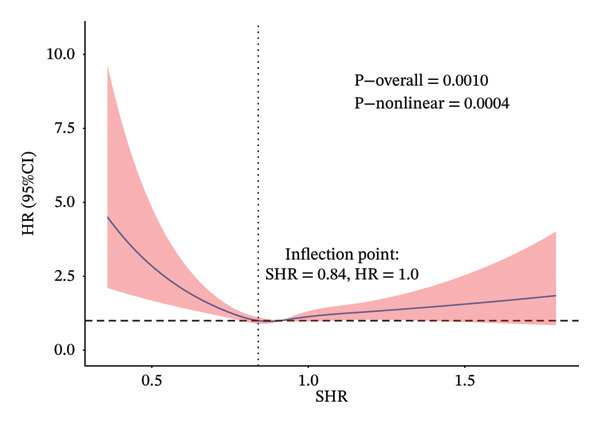
(b)

**TABLE 4:** SHR threshold effect on mortality.

	Adjusted HR (95% CI)	*p* value
ACM		
Total	1.76 (1.22, 2.53)	0.002

*Two-Piecewise Cox Model*		
Inflection: SHR = 0.81		
SHR < 0.81	0.77 (0.68, 0.88)	< 0.001
SHR ≥ 0.81	1.10 (1.05, 1.15)	< 0.001
CVM		
Total	0.82 (0.30, 2.26)	0.700

*Two-Piecewise Cox Model*		
Inflection: SHR = 0.84		
SHR < 0.84	0.73 (0.60, 0.90)	0.003
SHR ≥ 0.84	1.08 (1.00, 1.16)	0.045

*Note:* The model was adjusted for age, sex, race, body mass index, education, smoking, and family poverty income ratio, cardiovascular diseases, diabetes, cancer, blood pressure–lowering drugs, lipid‐lowering drugs, systolic blood pressure, eGFR.

Furthermore, SHR was classified into the two corresponding segments depending on the inflection point, and then the KM survival curve was conducted. These results revealed that there were significant differences in ACM (Figure 3(a)) and CVM (Figure 3(b)) between the two groups.

FIGURE 3
*K*–*M* survival analysis of ACM and CVM in the general population among the four SHR groups (Q1–Q4).

**Figure:**
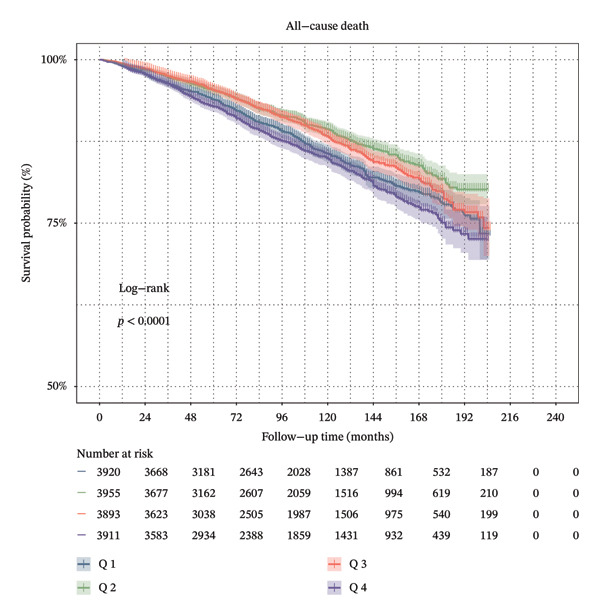
(a)

**Figure:**
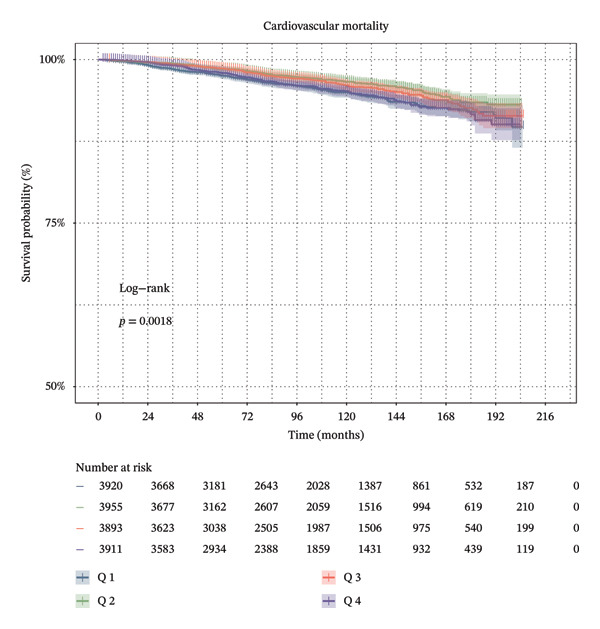
(b)

We evaluated the predictive power of SHR for ACM and CVM. A subgroup analysis was also performed.

By analyzing the ROC curves, we can see that SHR had an area under the curve (AUC) of 0.87 after 3 years, 0.88 after 5 years, and 0.89 after 10 years in relation to ACM (Figure 4(a)), and an AUC of 0.90, 0.91, and 0.90 after 10 years in relation to CVM (Figure 4(b)). Based on these results, the SHR may be a useful predictor of both ACM and CVM.

FIGURE 4The time‐dependent ROC curve of SHR used for indicating ACM and CVM.

**Figure:**
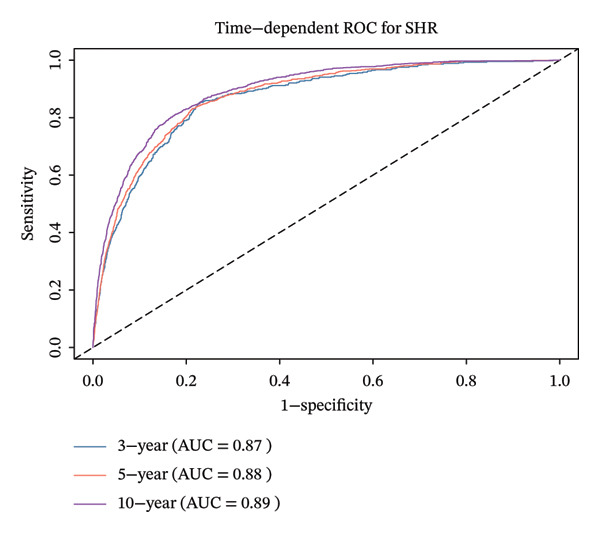
(a)

**Figure:**
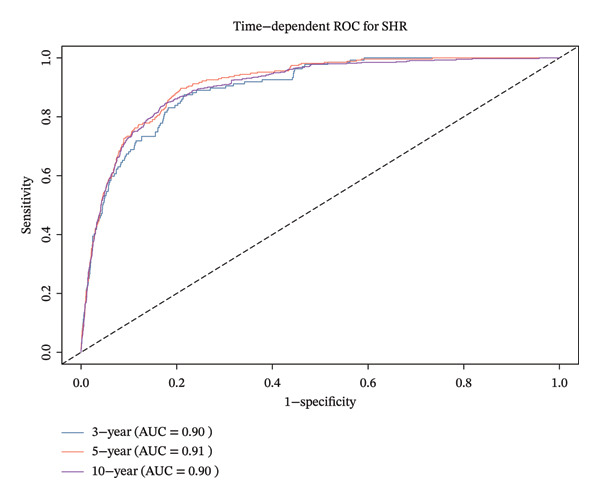
(b)

In various subgroups, SHR did not interact significantly with ACM (Figure 5(a)) and CVM (Figure 5(b)) when categorized by age, sex, race, BMI, education, smoking, and PIR.

FIGURE 5Subgroup analysis of the association between SHR and all‐cause mortality and CV mortality.

**Figure:**
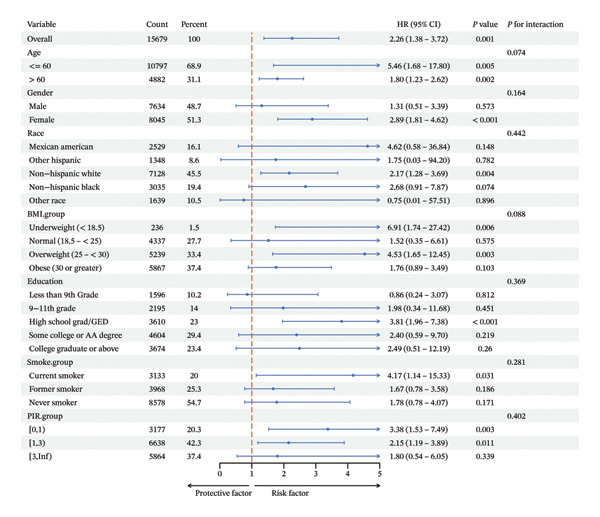
(a)

**Figure:**
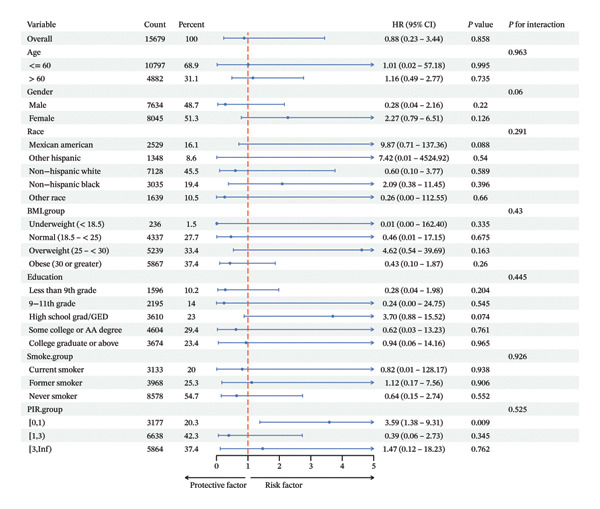
(b)

## 4. Discussion

This investigation uses the NHANES database to assess the SHR predictability for ACM and CVM in the general population. It is the first of its kind. The following are the main conclusions: first, SHR was found to be independently correlated with ACM and CVM in the general population; second, SHR and both forms of mortality showed a U‐shaped connection. In particular, when SHR is greater than 0.81, ACM is significantly higher, and when SHR is greater than 0.84, CVM is significantly higher; finally, SHR shows a suggestive predictive validity for both ACM and CVM in the general population.

SHR is a direct, straightforward, and measurable approach for assessing stress conditions, and its correlation with mortality has been extensively validated across many populations. Prior research has established that SHR is independently and nonlinearly linked to ACM and CVM in individuals with diabetes or prediabetes and chronic kidney disease or diabetic nephropathy [2, 9]. In addition, for critically ill patients, such as cardiac intensive care unit patients and sepsis patients, the SHR was strongly associated with mortality [10, 11]. Furthermore, the SHR acts as a valuable metric for predicting therapeutic outcomes. In individuals with heart failure worsened by sepsis, SHR serves as an independent prognostic indicator and exhibits a substantial U‐shaped correlation with ACM [5]. Major CV adverse events are more likely to happen in individuals with ST‐segment elevation myocardial infarction (STEMI) who have SHR. Additionally, it enhanced the thrombolysis in myocardial infarction risk score prediction efficiency in STEMI patients, particularly those with diabetes. A bad prognosis for patients with acute decompensated heart failure can be predicted by either an increase or a reduction in SHR [12]. Additionally, it enhanced the thrombolysis in the myocardial infarction risk score prediction efficiency in STEMI patients, particularly those with diabetes [12]. A bad prognosis for patients with acute decompensated heart failure can be predicted by either an increase or a reduction in SHR [13]. The significance of SHR in patient care can be ascertained from the aforementioned research.

The association between SHR and ACM, as well as CVM in the general population, remains ambiguous. This study may be the inaugural investigation to establish that SHR has a U‐shaped correlation with ACM and CVM in the general population, demonstrating strong predictive capability. The analysis of this study’s results, encompassing 15,679 people, established a U‐shaped link between the SHR and both ACM and CVM, following adjustments for age, gender, race, BMI, education, smoking, and PIR. Specifically, when SHR < 0.81, lower SHR was associated with a reduced risk of ACM, while when SHR > 0.81, a higher SHR was associated with a significantly increased risk of ACM. Similarly, for CV death, when SHR < 0.84, lower SHR was associated with a reduced risk of CVM, whereas when SHR > 0.84, higher SHR was associated with an increased risk of CVM. While the RCS analysis identified inflection points at 0.81 for ACM and 0.84 for CVM, these values should be interpreted as statistical approximations of the point at which the hazard ratio transitions from below to above 1, rather than as definitive clinical thresholds. RCS is a tool for describing nonlinearity, and any threshold derived from such models should be viewed as a reference point for further investigation, not as a rigid decision boundary. Previously, Yang et al. [3] discovered that SHR is linked to significant unfavorable cardiovascular events in individuals with acute coronary syndrome, Ding et al. [2] indicated that SHR correlated with ACM in patients with diabetes or prediabetes, while Cao et al. [9] identified a U‐shaped link between SHR and CVM in individuals suffering from chronic kidney disease and diabetic nephropathy. The above results may indicate that a mild increase in SHR under mild stress could be a protective mechanism of the body. However, when the SHR continues to rise, it may cause CV load and vascular damage, thereby possibly contributing to an increased risk of death.

The observed U‐shaped relationship, with inflection points at 0.81 for ACM and 0.84 for CVM, suggests a complex biological response to glycemic stress. The association with lower risk observed at moderately low SHR levels may represent an adaptive stress response, in which a mild increase in glucose provides essential fuel for vital organs (e.g., brain, immune cells) during periods of physiological challenge. This phenomenon is often referred to as “adaptive” or “benign” stress hyperglycemia [14, 15]. This transient state may enhance cellular resilience and facilitate recovery without causing significant harm.

In addition, after the adjustment for factors including age, gender, race, BMI, education, smoking, and PIR, the SHR was classified as either higher or lower SHR depending on inflection points. Both ACM and CVM showed a significant variation between the groups with greater and lower SHR, according to the KM survival curve. After the adjustment for age, sex, ethnicity, BMI, education, smoking, and PIR, the ROC curve used SHR as a continuous variable and showed that SHR is an excellent indicator of ACM and CVM at 3.5 and 10 years. This is similar to the findings of Tuersun et al. in psoriasis patients [16]. Further subgroup analyses showed that there was no statistical difference in the association between SHR, ACM, and CVM between different subgroups.

From a clinical perspective, the identification of these thresholds has significant implications for risk stratification in the general population. Our findings suggest that SHR could serve as a simple, readily available biomarker to identify individuals with an underlying maladaptive stress response who are at an increased long‐term mortality risk. For instance, individuals with an SHR > 0.84 could be identified as candidates for more intensive cardiovascular risk factor modification, including stricter blood pressure control, aggressive lipid management, and lifestyle interventions such as dietary optimization and regular exercise, even if they do not meet the traditional diagnostic criteria for diabetes. Conversely, an SHR below the threshold, particularly in the context of acute illness, might be interpreted as a favorable prognostic indicator. It is essential to emphasize, however, that these thresholds should not be used as standalone diagnostic tools but rather as adjuncts to comprehensive clinical assessment. By integrating both acute and chronic glycemic status, SHR offers a more nuanced view of an individual’s metabolic health and resilience than either fasting glucose or HbA1c alone. This may facilitate a shift toward more personalized preventive strategies, moving beyond a one‐size‐fits‐all approach to risk management. Further prospective studies are needed to determine whether interventions aimed at modulating the stress response or optimizing glycemic control in individuals with elevated SHR can effectively improve clinical outcomes.

The observed association between the SHR and ACM and CVM in the general population may be related to the following physiological mechanisms: (1) Monnier et al. found that by measuring the excretion rate of free 8‐iso prostaglandin F2alpha in 24‐h urine, a quantitative indicator of oxidative stress, its production would be significantly increased under hyperglycemia conditions [17]. (2) A higher SHR is associated with an increased risk of thrombosis Specifically, hyperglycemia can lead to an abnormal increase in the fibrinolytic inhibitor PAI‐1, resulting in a decrease in fibrinolytic capacity. When blood glucose decreases, PAI‐1 also decreases accordingly [18]. Furthermore, a high SHR is associated with an imbalance between endothelial dilation and contraction to shift toward contraction; that is, endothelial contraction factors (endothelin, angiotensin II, etc.) dominate [19]. Disorders of the fibrinolytic system and endothelial contraction dysfunction can both lead to abnormal activation of platelets, thereby triggering platelet aggregation and elevating the thrombosis risk and the occurrence of CV events [20]. (3) Studies have also shown that high SHR is associated with an abnormal increase in the stress hormones’ levels including catecholamines, glucagon, and cortisol [21, 22], which in turn leads to insulin resistance, elevated blood glucose levels, and exacerbated oxidative stress and endothelial damage, thereby potentially damaging the CV system [17]. In addition, high SHR may also be associated with aggravated CV injury and systemic inflammatory response by abnormally stimulating the renin–angiotensin–aldosterone system. Meanwhile, hyperglycemia mediates adipocyte hypertrophy and promotes the release of proinflammatory factors and insulin resistance, thereby leading to microvascular damage [23]. (4) Hyperglycemia is often accompanied by poor wound healing, increased risk of infection, weakened immunity, etc., increasing the risk of disease. Thus, the altered physiologic mechanisms described above collectively contribute to the observed increased risk of ACM and CVM.

This study demonstrated that SHR, which combines FPG and HbA1c, an easy‐to‐measure and obtainable indicator, may be a valid clinical indicator for assessing ACM and CVM in the general population and can help clinicians to quickly detect high‐risk populations and thus guide treatment. Thanks to the rigor of the NHANES survey process and the use of sampling weights, the reliability of the results of this study has increased.

Nevertheless, several limitations should be acknowledged. First, despite comprehensive adjustment for a wide range of demographic, socioeconomic, and clinical covariates, the possibility of residual confounding due to unmeasured factors (e.g., dietary habits, physical activity, genetic predisposition, or detailed medication adherence) cannot be entirely excluded, as is inherent to all observational studies. Second, this study employed a complete‐case analysis, which may introduce selection bias if missingness is not completely at random. Although we provided a detailed accounting of excluded participants in Figure 1 and compared baseline characteristics between included and excluded individuals in Supplementary Table 1—revealing only modest differences—the potential for bias cannot be fully ruled out. Third, the data were derived from the NHANES, which represents the noninstitutionalized U.S. population; therefore, the generalizability of our findings to other populations or settings may be limited. Future studies incorporating diverse cohorts from different geographic regions and ethnic backgrounds are warranted to validate and extend the applicability of our results.

## 5. Conclusion

This research revealed a U‐shaped connection between SHR and both ACM and CVM, with inflection points at 0.81 and 0.84, respectively. The SHR may serve as a useful indicator of ACM and CVM in the general population. These findings highlight the importance of the SHR in therapeutic practice.

## Author Contributions

Conceptualization and revision, Xin Li and Dexiang Xia; data analysis and writing Lei Zhang and Chang Shu; data curation, PengCheng Guo; supervision, WanCheng Guo and WenTao Hu; software: Rui Li.

## Funding

This work was supported by the National Natural Science Foundation of China (NSFC) (grant number 82120108005) and supported by the Postgraduate Innovative Project of Central South University (grant number 2022XQLH153).

## Ethics Statement

The NCHS Ethics Review Board approved the study protocol and obtained informed consent from all participants.

## Conflicts of Interest

The authors declare no conflicts of interest.

## Supporting Information

Supporting Table 1. Baseline characteristics of participants included versus excluded from the analytic sample.

## Supporting information


**Supporting Information** Additional supporting information can be found online in the Supporting Information section.

## Data Availability

The data used to support this study are available at https://www.cdc.gov/nchs/nhanes/.
