# Supplementary material for: Stress Hyperglycemia Ratio as a Predictor of All‐Cause and Cardiovascular Mortality in the General Population: Insights From NHANES 2001–2018
Source: Int J Endocrinol. 2026 Apr 22;2026:9929815. doi: 10.1155/ije/9929815 (PMC13101950; doi:10.1155/ije/9929815)
Supplement: Supplementary file 1 — Supporting Information Additional supporting information can be found online in the Supporting Information section. [file IJE-2026-9929815-s001.docx]

**Supplementary Table 1. Baseline characteristics of participants included versus excluded from the analytic sample**

| Characteristic | Overall | Data after censoring | p | SMD |
| --- | --- | --- | --- | --- |
| Unweighted N | 50,201 | 15,679 |  |  |
| Age (years) | 47.0 (16.9) | 47.0 (16.8) | 0.897 | 0.001 |
| Male (%) | 24,204 (48) | 7,634 (49) | <0.001 | 0.011 |
| Race (%) |  |  | 0.009 | 0.029 |
| Mexican American | 8,311 ( 8.3) | 2,529 ( 8.2) |  |  |
| Other Hispanic | 4,212 ( 5.3) | 1,348 ( 4.9) |  |  |
| Non-Hispanic White | 22,090 (67.8) | 7,128 (68.9) |  |  |
| Non-Hispanic Black | 10,606 (11.3) | 3,035 (10.7) |  |  |
| Other Race | 4,982 ( 7.3) | 1,639 ( 7.3) |  |  |
| BMI.group (%) |  |  | 0.016 | 0.016 |
| Normal(18.5 to <25) | 13,123 (29.3) | 4,337 (29.0) |  |  |
| Obese(30 or greater) | 17,266 (35.8) | 5,867 (36.4) |  |  |
| Overweight(25 to <30) | 15,799 (33.3) | 5,239 (33.0) |  |  |
| Underweight(<18.5) | 768 ( 1.6) | 236 ( 1.5) |  |  |
| Education (%) |  |  | <0.001 | 0.04 |
| Less Than 9th Grade | 5,906 ( 6.0) | 1,596 ( 5.3) |  |  |
| 9-11th Grade | 7,338 (11.0) | 2,195 (10.6) |  |  |
| High School Grad/GED | 11,636 (23.8) | 3,610 (23.4) |  |  |
| Some College or AA degree | 14,296 (31.2) | 4,604 (31.3) |  |  |
| College Graduate or above | 10,929 (28.1) | 3,674 (29.4) |  |  |
| PIR.group (%) |  |  | 0.151 | 0.01 |
| [1,3) | 19,319 (36.7) | 6,638 (36.7) |  |  |
| [3,Inf) | 16,883 (49.2) | 5,864 (49.6) |  |  |
| [0,1) | 9,429 (14.0) | 3,177 (13.7) |  |  |
| Smoking status (%) |  |  | 0.152 | 0.011 |
| Never smoker | 27,419 (54.0) | 8,578 (54.2) |  |  |
| Former smoker | 12,293 (25.1) | 3,968 (25.4) |  |  |
| Current smoker | 10,432 (20.9) | 3,133 (20.4) |  |  |
| SBP | 121.72 (17.50) | 121.44 (17.11) | <0.001 | 0.016 |
| eGFR | 97 (21) | 97 (21) | 0.823 | 0.001 |
| lipid_med | 9,720 (81.8) | 3,271 (81.1) | <0.001 | 0.017 |
| HTN_med | 13,509 (76.8) | 4,261 (76.7) | 0.88 | 0.001 |
| CVD (%) | 5,774 (91.2) | 1,721 (91.2) | 0.618 | 0.002 |
| Diabetes.group (%) |  |  | <0.001 | 0.027 |
| diabetes | 8,635 (14.6) | 3,094 (14.7) |  |  |
| normal | 27,078 (45.5) | 6,069 (44.2) |  |  |
| prediabetes | 14,488 (39.9) | 6,516 (41.1) |  |  |
| Cancer (%) | 4780 (90.6) | 1460 (90.7) | 0.701 | 0.002 |
